# Supplementary material for: Resolving temporal threat uncertainty by observational learning involves the amygdala, hippocampus and anterior insula
Source: Transl Psychiatry. 2026 Sep 26;16:488. doi: 10.1038/s41398-026-04492-7 (PMC13615975; doi:10.1038/s41398-026-04492-7)
Supplement: Supplementary file 1 [file 41398_2026_4492_MOESM1_ESM.docx]

**Supplementary Text 1: Other Expectation**

A 3x2 repeated measurement ANOVA revealed a significant influence of the condition (F = 26.685, df = 2, p < 0.001), cue/condition-onset (F = 14.936, df = 1, p < 0.001) and their interaction (F = 8.325, df =2, p < 0.001). Post-hoc t-tests of that interaction showed an increased cue-prominence in both U (t= 4.751, df = 19, p = 0.00014, p_Holm_ = 0.00042) and N (t= 3.434, df = 19, p = 0.003, p_Holm_ = 0.006) as compared to P.

**Supplementary Text 2: SCR**

13 subjects were included into the analysis after data-quality assessment in the observation phase. Only the factor cue/condition-onset significantly influenced SCR (F = 17.309, df = 1, p = 0.001) but not condition (F = 1.438, df = 2, p = 0.257) or the interaction of condition and cue/condition-onset (F = 0.631, df = 2, p = 0.541). A post-hoc test revealed an increased response to condition-onsets as compared to cues among all conditions (t = 4.16, df = 12, p = 0.001). Only a descriptive difference (P (mean ± SD) = 0.401 ± 0.948; U (mean ± SD) = 0.195 ± 0.794; N (mean ± SD) = 0.197 ± 0.934) among the responses to predictable pain as compared to both unpredictable (t = 0.896, df = 12, p = 0.388) and no pain (t = 0.067, df = 12, p = 0.516) was revealed.

In the expression phase 15 subjects were included after data-quality assessment. No significant effect or interaction-effect of condition or cue/condition-onset SCR was found (condition: F = 2.285, df = 2, p = 0.120; cue/condition-onset: F = 3.891, df = 1, p = 0.069; conditionXcue/condition-onset: F = 20.083, df = 2, p = 0.921).

| **Table S1. Experiment 1:** repeated measurement ANOVA. Threat Expectation | | | | | | |
| --- | --- | --- | --- | --- | --- | --- |
| **Cases** | **Sum of Squares** | **df** | **Mean Square** | **F** | **p** | **η²_p_** |
| Within Subjects Effects | | | | | | |
| **Condition (P,U,N)** | 6.99 | 2 | 3.495 | 13.594 | < 0.001 | 0.475 |
| Residuals | 7.713 | 30 | 0.257 |  |  |  |
| **Cue/Condition(mean)** | 22.248 | 1 | 22.248 | 123.337 | < 0.001 | 0.892 |
| Residuals | 2.706 | 15 | 0.18 |  |  |  |
| **Condition X Cue/ Condition(mean)** | 2.587 | 2 | 1.294 | 12.397 | < 0.001 | 0.452 |
| Residuals | 3.131 | 30 | 0.104 | 13.594 |  | 0.475 |

**Table S1 legend.** Table S1 reports the detailed results of the behavioral effect (threat expectation) rmANOVA (3x2: Condition: P, U, N; Timepoint: cue, mean(condition-onset,condition-offset)) of Experiment 1. We report sum of squares, degrees of freedom, mean square, F-values, p and an estimation of the effect sizes (partial η²) for the main effects of condition and timepoint and the interaction effect.

**Table S2.**  **Experiment 1:** Paired-sample t-tests. Learned cue-prominence after observation phase.

| **Variable 1** | **Variable 2** | ***df*** | ***T*** | ***p*** | ***p_Holm_*** | **Cohen’s d** |
| --- | --- | --- | --- | --- | --- | --- |
| Cue-Prominence P | Cue-Prominence N | 15 | 4.653 | 0.0003126 | 0.0009378 | 1.163 |
| Cue-Prominence U | Cue-Prominence N | 15 | 2.582 | 0.021 | 0.021 | 0.645 |
| Cue-Prominence P | Cue-Prominence U | 15 | 2.677 | 0.017 | 0.034 | 0.669 |

**Table S2 legend.** Table S2 reports the posthoc paired-sample t-tests conducted to analyze the significant interaction effect of the rmANOVA in Experiment 1. Note that the cue-prominence is here calculated as cue – mean(condition-onset, condition-offset). We compare the learned cue-prominence between all three conditions. Degrees of freedom, T, p, p corrected for multiple comparisons (Bonferroni-Holm) and an estimation of effect size (Cohen’s d) are reported.

**Table S3.** **Experiment 1:** Behavioral outcome measures

| **Variable** | **Descriptives** |  |
| --- | --- | --- |
| Cue-Prominence P (mean ± SD) | 133.5 ± 44.1 |  |
| Cue-Prominence U (mean ± SD) | 101.7 ± 52.2 |  |
| Cue-Prominence N (mean ± SD) | 53.6 ± 55.8 |  |

**Table S3 legend.** Table S3 reports the descriptive results of Experiment 1. We report the calculated cue-prominence for each condition (P, U, N). Note that the cue-prominence is here calculated as cue – mean(condition-onset, condition-offset t).

| **Table S4. Experiment 2:** repeated measurement ANOVA. Threat Expectation | | | | | | |
| --- | --- | --- | --- | --- | --- | --- |
| **Cases** | **Sum of Squares** | ***df*** | **Mean Square** | ***F*** | ***p*** | ***η²_p_*** |
| Within Subjects Effects | | | | | | |
| **Condition(P,U,N)** | 8.988 | 2 | 4.494 | 24.917 | <.001 | 0.531 |
| Residuals | 7.936 | 44 | 0.18 |  |  |  |
| **Cue/Condition(mean)** | 9.257 | 1 | 9.257 | 52.736 | < .001 | 0.706 |
| Residuals | 3.862 | 22 | 0.176 |  |  |  |
| **Condition X Cue/Condition(mean)** | 1.938 | 2 | 0.969 | 13.835 | < .001 | 0.386 |
| Residuals | 3.082 | 44 | 0.07 |  |  |  |

**Table S4 legend.** Table S4 reports the detailed results of the behavioral effect (threat expectation) rmANOVA (3x2: Condition: P, U, N; Timepoint: cue, mean(condition-onset, condition-offset)) of Experiment 2. We report sum of squares, degrees of freedom, mean square, F-values, *p* and an estimation of the effect sizes (*partial η²)* for the main effects of condition and timepoint and the interaction effect.

**Table S5.**  **Experiment 2:** Paired-sample t-tests. Learned cue-prominence after observation phase.

| **Variable 1** | **Variable 2** | ***df*** | ***T*** | ***p*** | ***p_Holm_*** | **Cohen’s d** |
| --- | --- | --- | --- | --- | --- | --- |
| Cue-Prominence P | Cue-Prominence N | 22 | 4.497 | 0.0001793 | 0.0005379 | 0.352 |
| Cue-Prominence U | Cue-Prominence N | 22 | 3.671 | 0.001 | 0.002 | 0.288 |
| Cue-Prominence P | Cue-Prominence U | 22 | 1.631 | 0.117 | 0.117 | 0.185 |

**Table S5 legend.** Table S5 reports the posthoc paired-sample t-tests conducted to analyze the significant interaction effect of the rmANOVA in Experiment 2. Note that the cue-prominence is here calculated as cue – mean(condition-onset, condition-offset). We compare the learned cue-prominence between all three conditions. Degrees of freedom, *T, p, p* corrected for multiple comparisons (Bonferroni-Holm) and an estimation of effect size (Cohen’s d) are reported.

**Table S6.** **Experiment 2:** Behavioral outcome measures

| **Variable** | **Descriptives** |  |
| --- | --- | --- |
| Cue-Prominence P (mean ± SD) | 75.5 ± 50.5 |  |
| Cue-Prominence U (mean ± SD) | 70.5 ± 52.1 |  |
| Cue-Prominence N (mean ± SD) | 19.4 ± 32.5 |  |

**Table S6 legend.** Table S6 reports the descriptive results of Experiment 2. We report the calculated cue-prominence for each condition (P, U, N). Note that the cue-prominence is here calculated as cue – mean(condition-onset, condition-offset).
